# Rice–crayfish farming mode drives distinct soil properties and ecological assembly of soil microbiome

**DOI:** 10.1128/spectrum.03832-25

**Published:** 2026-08-06

**Authors:** Liang Peng, Li-li Dai, Ling Tao, Gu Li, Jian-qiang Zhu, Hui Zhang

**Affiliations:** 1 Yangtze River Fisheries Research Institute, Chinese Academy of Fishery Sciences 499154, Wuhan, China; 2 College of Agriculture, Yangtze University 47897, Jingzhou, China; National Taiwan University, Taipei, Taiwan

**Keywords:** Rice–crayfish farming mode, soil properties, microbiome, ecological assembly

## Abstract

Rice–crayfish farming (RCF) system represents an effective ecological agricultural model characterized by the cyclical spatiotemporal integration of rice farming and crayfish aquaculture. However, the effects of farming mode and stage on soil microbial community structure over time remain insufficiently explored. In this study, we investigated taxonomic and functional changes in soil microbiomes and their associations with soil nutrient fertility in both RCF and rice monoculture (RM) systems. Our findings demonstrated that RCF significantly increased soil pH, total nitrogen (TN), and soil organic carbon (SOC) compared to RM across multiple growth stages (*P* < 0.05). Two-way analysis of variance showed that both mode and stage affected the Chao1 index, while the Shannon index was only affected by stage. Microbial community analysis revealed clear structural differences between the two systems (*P* < 0.001). Functional prediction indicated lower chemoheterotrophy but higher photoheterotrophy, aromatic degradation, and sulfur cycling in RCF, along with reduced nitrogen cycling function. Co-occurrence network analysis further showed a longer average path length and higher modularity in RCF than in RM. Modules 3 and 6 in RCF were positively correlated with pH, TN, and SOC. Overall, RCF stabilizes the soil environment and selects for specific functionally sensitive taxa, thereby promoting the formation of a highly modular microbial network, which ultimately maintains the synergistic stability of soil nutrients and the microbial community.

**IMPORTANCE:**

The present study comprehensively compared two different farming modes in terms of their soil microbiome structures and the associations between the microbiomes and soil nutrient fertility. Rice–crayfish farming (RCF) model-specific microbial taxa were identified, and their modularity was found in RCF. These findings provide valuable insights into microbial community responses and regulation in ecological agriculture, establishing a robust microbiological foundation for optimizing rice-aquatic animal integrated farming management and advancing sustainable agricultural practices.

## INTRODUCTION

Rice–aquatic animal integrated farming (RAA) is a prominent ecological agricultural system in Asia with a history spanning over two millennia (1). This system optimizes the utilization of diverse resources, including water, land, organisms, and nutrients, while significantly reducing dependence on chemical fertilizers and pesticides (2, 3). RAA has been shown to enhance rice productivity (4) and improve the environmental quality of the ecosystems (5, 6). Rice–crayfish farming (RCF) has emerged as a particularly lucrative practice, leading to its rapid expansion in China’s waterlogged regions (7). By 2023, the RCF system covered 1.68 million hectares and produced 2.75 million tons of crayfish, which constitutes 85.76% of the total RCF area and 87.00% of China’s crayfish output (8). Despite its significance in grain production and economic viability, RCF has not been extensively studied beyond these aspects. Therefore, there is an urgent need for a comprehensive evaluation of RCF to better understand its ecological impacts and address existing research gaps.

In the RCF model, a complex interplay exists among crayfish cultivation, plant growth, microbial communities, and environmental factors (9). Microorganisms play crucial roles that are essential for nutrient cycling, biogeochemical processes, and the sustainability of cultivated organisms. For example, soil microorganisms significantly contribute to the cycling of nitrogen and phosphorus, as well as energy flow, and can potentially mitigate eutrophication pollution (10, 11). Moreover, microorganisms involved in carbon transformation can significantly influence the dynamics of organic matter in paddy soils (12). Several studies have examined the effects of the RCF model on the soil microbiome (13–17). The introduction of crayfish (13, 14), artificial feeds (15, 16), and reduced fertilizer application (17, 18) has been shown to cause substantial shifts in soil microbial communities within the RCF system. Research also indicates that RCF can markedly alter soil functional genes, increasing the abundance of genes related to greenhouse gas emissions, antibiotic resistance, and human pathogens (19). Furthermore, the overutilization of paddy fields may deplete soil microorganisms involved in nitrogen cycling while enriching those associated with the carbon cycle, thereby disrupting soil microbial ecology (20). In RCF, microorganisms present in water, unique to this model, may enhance crayfish growth and improve water quality (21, 22). However, current research often examines rice cultivation and crayfish farming separately, artificially dividing this integrated system. This fragmented approach disrupts the annual cycle, severs critical connections between the two phases, and limits our understanding of nutrient dynamics.

In this study, we employed 16S rRNA high-throughput sequencing to comprehensively assess taxonomic shifts within soil microbiomes and their responses to farming practices and stages at seven time points across two farming systems: the RCF and rice monoculture (RM). Soil samples were collected from both systems at seven time points throughout the entire cultivation cycle. The objectives of our study were to (i) evaluate the successional dynamics of soil microbial community structure and function over a temporal gradient, (ii) analyze the interactive effects of cultivation regimes and stages, and (iii) identify critical phase-specific drivers and sensitive microbial taxa responsible for community differentiation. This is the first study to apply high-throughput sequencing combined with co-occurrence network analysis conducted over the full cycle of integrated farming to comprehensively evaluate its impact on soil microbial communities.

## MATERIALS AND METHODS

### Experimental design

**Fig 1:**
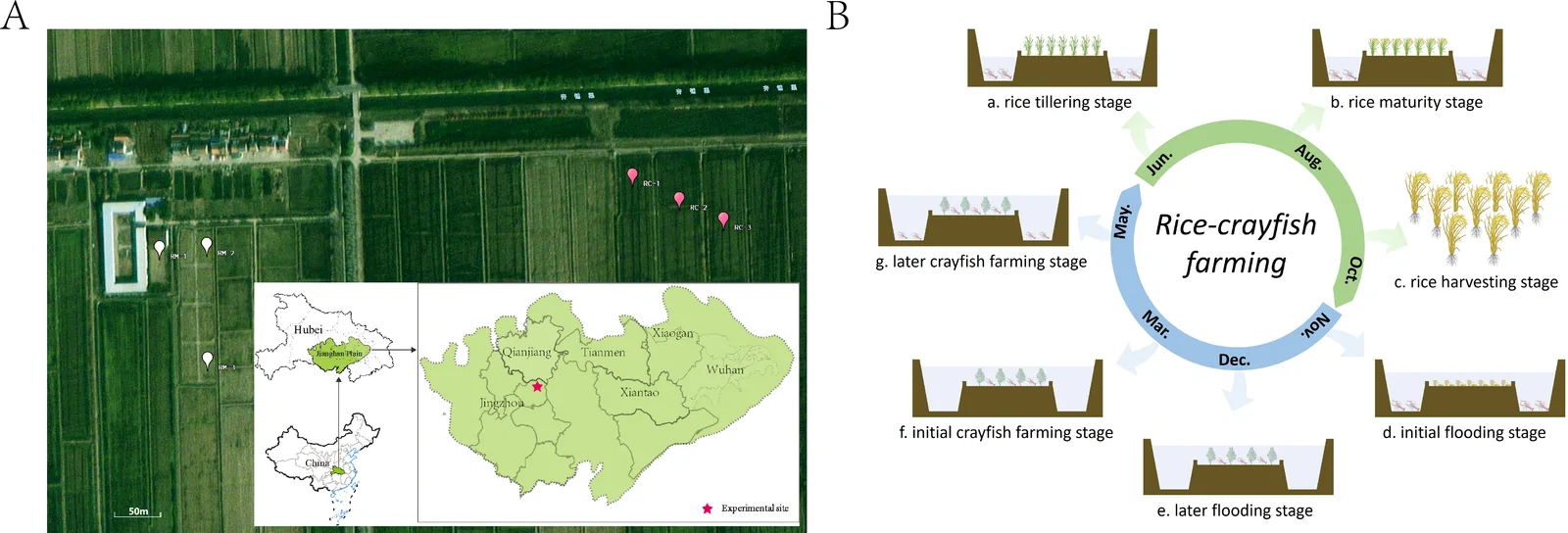
Sampling site of the experiment (**A**) and the typical temporal cycle of the rice–crayfish farming (**B**).

The experiment was conducted from July 2021 to May 2022 in a 4-year rice–crayfish farming paddy field at Sanhu Farm, Jiangling County, Hubei Province (30°19′ N, 112°54′ E; Fig. 1A). This region experiences a northern subtropical monsoon climate, with an average annual temperature of 16°C and an annual precipitation of 1,100 mm. The annual sunshine duration ranges from 1,827 to 1,897 h, and the frost-free period lasts between 246 and 262 days. To ensure consistency, three typical standardized RCF and RM fields, each averaging 667 m^2^ in area, were selected.

The RCF fields consisted of a rice planting area and an annular ditch measuring 2 m in width and 1.5 m in depth. The rice variety “Shen Liang You 3117” (*Oryza sativa L*.) was planted in June with a spacing of 16 × 30 cm. Fertilizer application was consistent with that used in the RM fields and included 10 kg/667 m^2^ of urea and 15 kg/667 m^2^ of phosphorus. Rice was harvested in early October, and the rice straw was retained in the field. Subsequently, the paddy field was flooded to a depth of 30 cm for crayfish (*Procambarus clarkii*) cultivation. During this period, the RM fields were maintained as winter-flooded paddy fields with a shallow water layer. Aquatic plants (*Elodea nuttallii*) were transplanted into the rice planting area in December, and crayfish larvae were introduced to the field for feeding in early March. The density of crayfish larvae introduced was approximately 25 kg/667 m^2^, and the average feed input was approximately 125 kg/667 m^2^. Except for crayfish cultivation, RCF management was consistent with that of RM.

### Soil sampling

Surface soil samples (0–20 cm) were collected from each paddy field at multiple time points: (a) July 21, (b) September 17, (c) October 22, (d) December 18, (e) January 20, (f) March 15, and (g) May 17. These sampling dates correspond to different farming stages: (a) rice tillering, (b) rice maturity, (c) rice harvesting, (d) initial flooding, (e) later flooding, (f) initial crayfish farming, and (g) later crayfish farming (Fig. 1B). At each sampling time, topsoil (0–20 cm depth) was collected using a stainless-steel core sampler within the rice planting area. A five-point sampling method following an S-shaped pattern was employed within each plot to ensure spatial representativeness. After removing visible stones and plant residues, the five soil cores were thoroughly mixed in equal proportions to form one composite sample. A total of six composite samples (two treatments × three replicates) were obtained at each sampling stage.

### Physicochemical analysis

All soil samples were divided into two portions. One portion was stored at –80°C for subsequent DNA extraction. After air drying, the other portion was used to determine physicochemical properties, including pH, moisture content, total nitrogen (TN), soil organic carbon (SOC), ammonia nitrogen (NH_4_^+^-N), and dissolved organic carbon (DOC). pH was measured using a glass electrode pH meter at a soil-to-water ratio (wt/vol) of 1:2.5. Moisture content was determined by the drying method. TN was measured using the Kjeldahl method. SOC was oxidized with potassium dichromate and quantified by titration with ferrous sulfate. NH_4_^+^-N was spectrophotometrically determined after extraction with a 1 mol·L^–1^ potassium chloride solution. DOC was extracted at a soil-to-water ratio (wt/vol) of 1:2.5 and measured using the potassium dichromate oxidation spectrophotometric method.

### DNA extraction and PCR amplification

Microbial community genomic DNA was extracted from soil samples using the TGuide S96 Magnetic Soil/Stool DNA Kit (Tiangen Biotechnology Co., Ltd., Beijing, China) according to the manufacturer’s instructions. The quality and quantity of the extracted DNA were assessed by electrophoresis on a 1.8% agarose gel. DNA concentration and purity were measured using a NanoDrop 2000 UV-Vis spectrophotometer (Thermo Scientific, Wilmington, USA). The hypervariable V3–V4 region of the bacterial 16S rRNA gene was amplified using the primer pairs 338F (5′-ACTCCTACGGGAGGCAGCA-3′) and 806R (5’- GGACTACHVGGGTWTCTAAT-3′). PCR products were verified by agarose gel electrophoresis and then purified using the Omega DNA Purification Kit (Omega, Inc.). The purified PCR products were pooled, and paired-end sequencing (2 × 250 bp) was performed on the Illumina NovaSeq 6000 platform (Beijing Biomarker Technology Co., Ltd., Beijing, China).

A total of 3,242,678 pairs of raw reads were obtained from the 42 samples. After paired-end quality control and merging, 2,950,928 clean reads remained, averaging 70,260 clean reads per sample. These clean reads were then subjected to feature classification to generate amplicon sequence variants (ASVs) using DADA2. ASVs with counts greater than three that appeared in more than three samples were retained. Taxonomy annotation of the ASVs was performed using the naive Bayes classifier in QIIME2, based on the Silva v138 database, with a confidence threshold of 70%.

### Data analysis

Differences in soil properties, microbial *α*-diversity, and predicted functions were assessed using the paired Wilcoxon rank sum test. Microbial *α*-diversity was evaluated using the abundance-based coverage estimator (ACE), Shannon index, and phylogenetic diversity whole-tree (PD-whole tree) index at the ASV level. A two-way analysis of variance (ANOVA) was performed to examine the effects of farming mode and stage on *α*-diversity and predicted functions, utilizing the “bruceR” package. Principal coordinate analysis (PCoA) was employed to visualize the microbial community composition, which was further quantified using permutational multivariate analysis of variance (PERMANOVA) with 999 permutations. The Functional Annotation of Prokaryotic Taxa (FAPROTAX) database was used to annotate and assess the predicted functions of microbial communities involved in soil biogeochemical cycles (23).

To identify the core microbiota that were most sensitive to the farming stages within each farming mode, we defined “Sensitive ASVs” following the approach described by Hartman et al. (24). Briefly, ASV abundances that significantly varied among the stages were identified using the “edgeR” package. Subsequently, indicator species analysis was performed with the “indicspecies” package to identify the ASVs positively associated with specific stages. The ASVs identified by both methods were then intersected and designated as “Sensitive ASVs.” ASVs identified by indicator species analysis were visualized using the bipartite package. A weighted bipartite graph was constructed to establish the association network between ASVs and groups. Furthermore, co-occurrence networks for indicator species were constructed based on Spearman rank correlations between ASVs, visualizing positive and significant correlations (*r* > 0.7 and *P* < 0.01). Redundancy analysis (RDA) was used to determine the correlations between the soil microbial community and soil properties.

All data analyses were performed using R software (version 4.4.0). Alpha diversity (ACE, Shannon, PD-whole tree), beta diversity (PCoA and PERMANOVA), and RDA were conducted using the “vegan” package in R. The co-occurrence network was visualized with the Fruchterman-Reingold layout using 100 permutations in Gephi 0.9.2. The sampling map was created using QGIS 3.28 Firenze.

## RESULTS

### Variations of the soil properties

**Fig 2:**
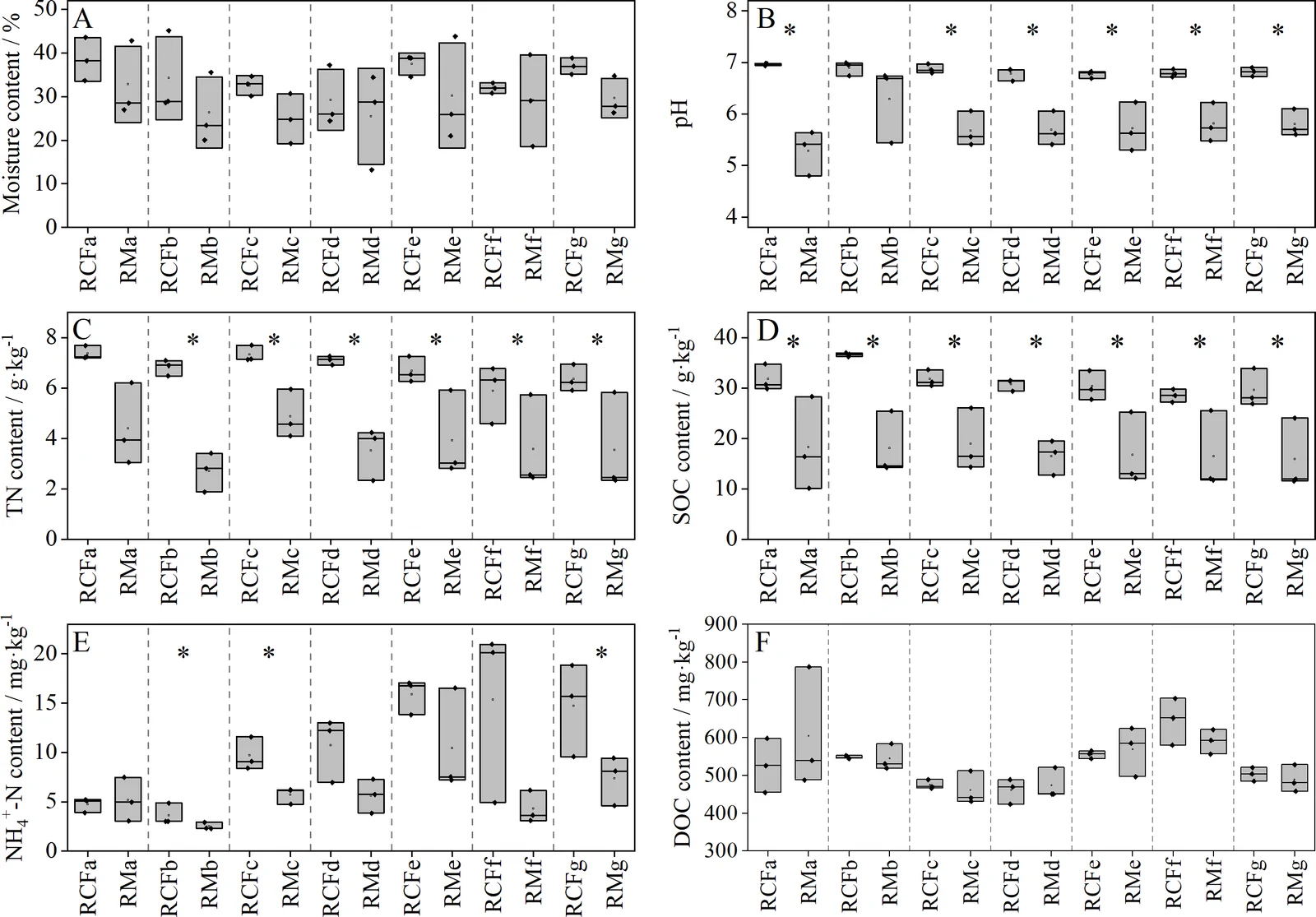
Dynamics of changes in the physicochemical properties of paddy soil across the RCF and RM groups throughout the entire farming stage ([**A**] Moisture content; [**B**] pH; [**C**] TN content; [**D**] SOC content; [**E**] NH4-N content; [**F**] DOC content). An asterisk indicates significant differences between the RCF and RM groups at specific farming stages with the Wilcoxon signed-rank test (*P* < 0.05).

Most soil properties differed significantly between the RCF and RM treatments (Fig. 2). Specifically, RCF exhibited significantly higher pH, TN, SOC, and NH_4_^+^-N contents than RM at multiple stages (e.g., stages b, c, and g). Notably, the soil pH, TN, and SOC content in RCF were markedly higher than those in RM across the five distinct periods (*P* < 0.05). RM showed substantial fluctuations in pH, TN, and SOC content, whereas the RCF exhibited less variation over the different periods. In both RCF and RM, NH_4_^+^-N content increased from stage b to stage e, with RCF maintaining significantly higher levels than RM during stages b, c, and g. However, no significant difference was observed in the DOC content between the two treatments.

Spearman correlation analysis indicated that TN and SOC contents were strongly correlated with soil pH and moisture content (*P* < 0.05; Table 1). Furthermore, TN was positively correlated with SOC, while pH was positively correlated with moisture content (*P* < 0.05).

**TABLE 1:** Spearman correlations between soil properties^*a*^

Item	Moisture	pH	TN	SOC	DOC	NH_4_^+^-N
NH_4_^+^-N	0.05	−0.13	−0.03	−0.12	−0.06	1.00
DOC	0.18	−0.02	−0.07	−0.05	1.00	
SOC	0.37*	0.77**	0.91**	1.00		
TN	0.39*	0.72**	1.00			
pH	0.33*	1.00				
Moisture	1.00					

^*a*^ The asterisk indicates a significant correlation between the two parameters. * *P* < 0.05, ** *P* < 0.01, the same as below.

### Dynamics of the diversity and composition of soil microbial communities

A total of 7,343 ASVs were obtained from all samples and assigned to 33 phyla. Among these, 368 ASVs (5.01%) were detected in at least half of the samples. Rarefaction analysis indicated that the detected ASVs for the soil microbiome reached a plateau, suggesting that most of the observed ASV richness was captured.

**Fig 3:**
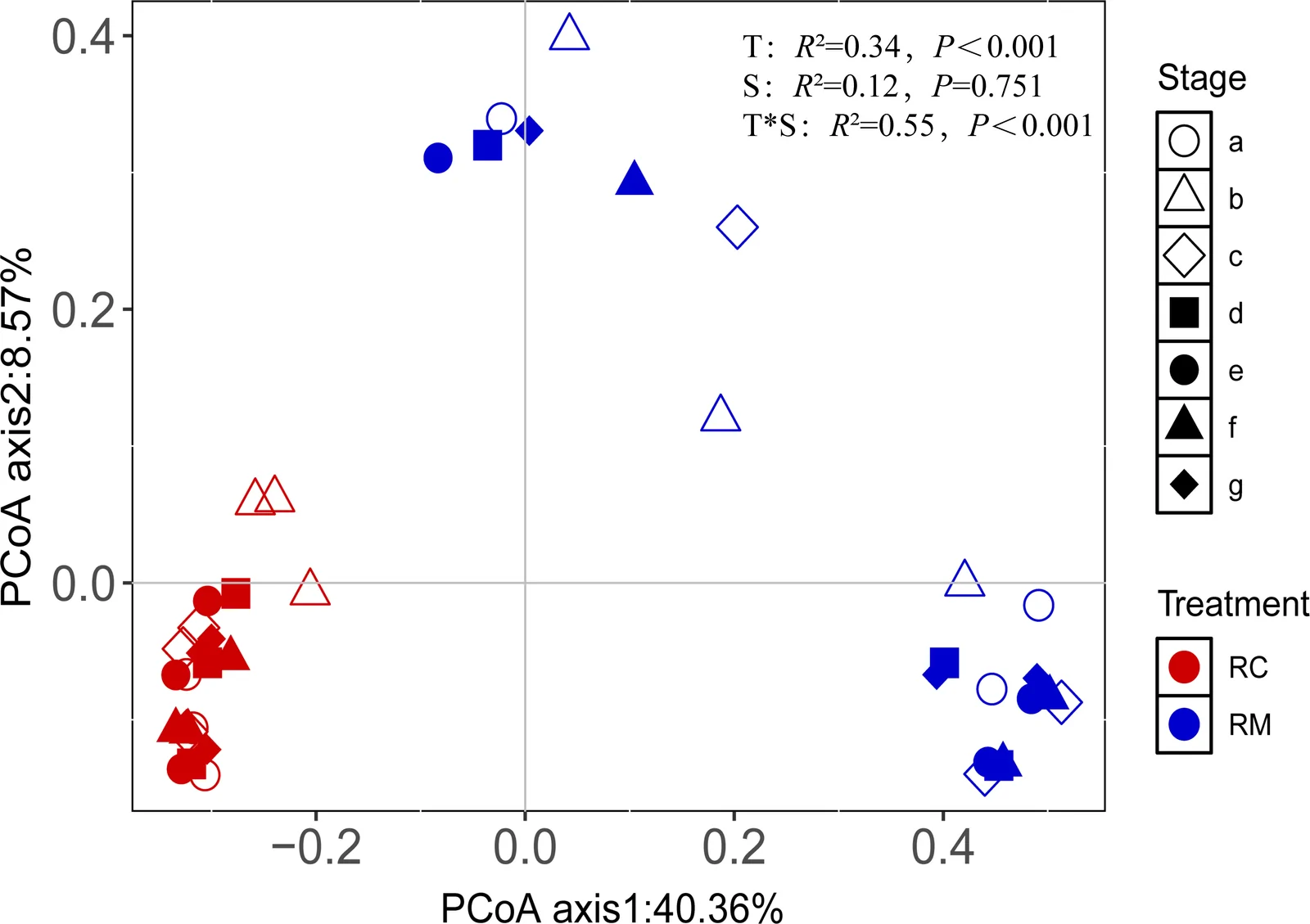
Principal coordinate analysis (PCoA) of soil microbial community.

Two-way ANOVA showed that the Chao1 index was significantly affected by farming mode and stage (Table 2). Additionally, Shannon indices were influenced only by the farming stage (*P* < 0.05). PCoA and PERMANOVA revealed a significant difference in the soil microbial structure between the two farming modes (*P* < 0.001; Fig. 3), while the effect of farming stage on soil microbial structure in RCF was also significant (*P* < 0.001).

**TABLE 2:** Two-way ANOVA examining the effects of farming mode and stage on microbial diversity indices^*a*^

	Mode (M)		Stage (S)		M × S	
	*F*	*P*	*F*	*P*	*F*	*P*
Chao1	4.765	0.0376*	11.02	<0.001**	0.175	0.9814
Shannon	1.851	0.1845	5.162	0.0011**	0.196	0.9753
Pielou	0.19	0.666	0.764	0.605	0.572	0.749

^*a*^ The asterisk indicates a significant difference between the two modes or stages. * *P* < 0.05, ** *P* < 0.01.

**Fig 4:**
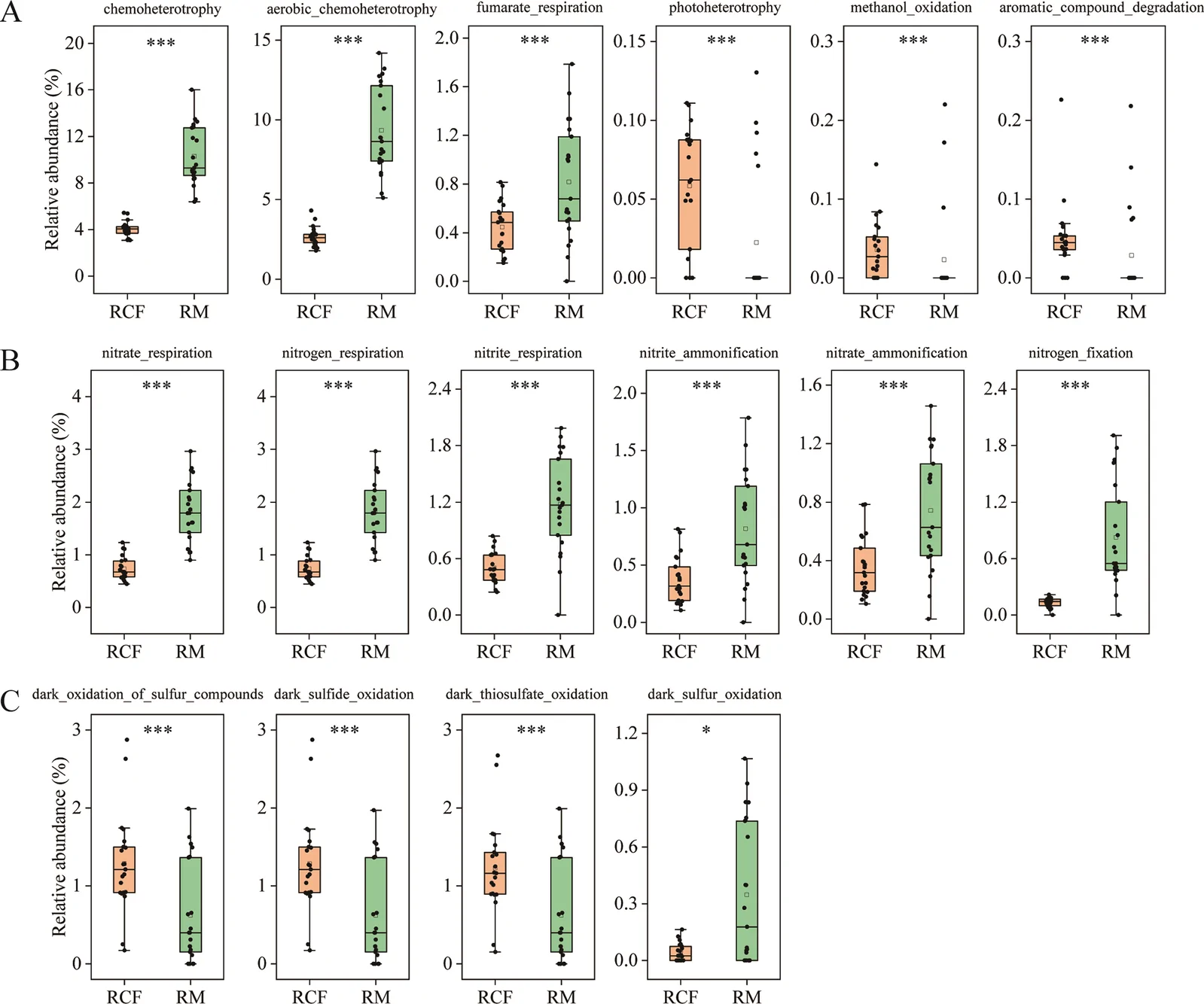
The predicted functions between two farming modes. (**A**) carbon cycling-related functions; (**B**) nitrogen cycling-related functions; (**C**) sulfur cycling-related functions. * *P* < 0.05 and *** *P* < 0.001.

FAPROTAX analysis of the microbial community revealed differences in the relative abundance of functions involved in biogeochemical cycling. The results showed that some carbon cycling-related functions (e.g., chemoheterotrophy, aerobic chemoheterotrophy, and fumarate respiration) were significantly lower in RCF than in RM, whereas other carbon cycling-related functions (e.g., photoheterotrophy, aromatic compound degradation, and methanol oxidation) were significantly higher in RCF compared to RM. Additionally, RCF exhibited lower nitrogen cycling-related functions (e.g., nitrate respiration, nitrogen respiration, nitrite respiration, nitrite ammonification, and nitrate ammonification), but higher sulfur cycling-related functions (e.g., dark oxidation of sulfur compounds, dark sulfide oxidation, dark thiosulfate oxidation, and dark sulfur oxidation) relative to RM (Fig. 4).

### Sensitive ASVs and co-occurrence network

**Fig 5:**
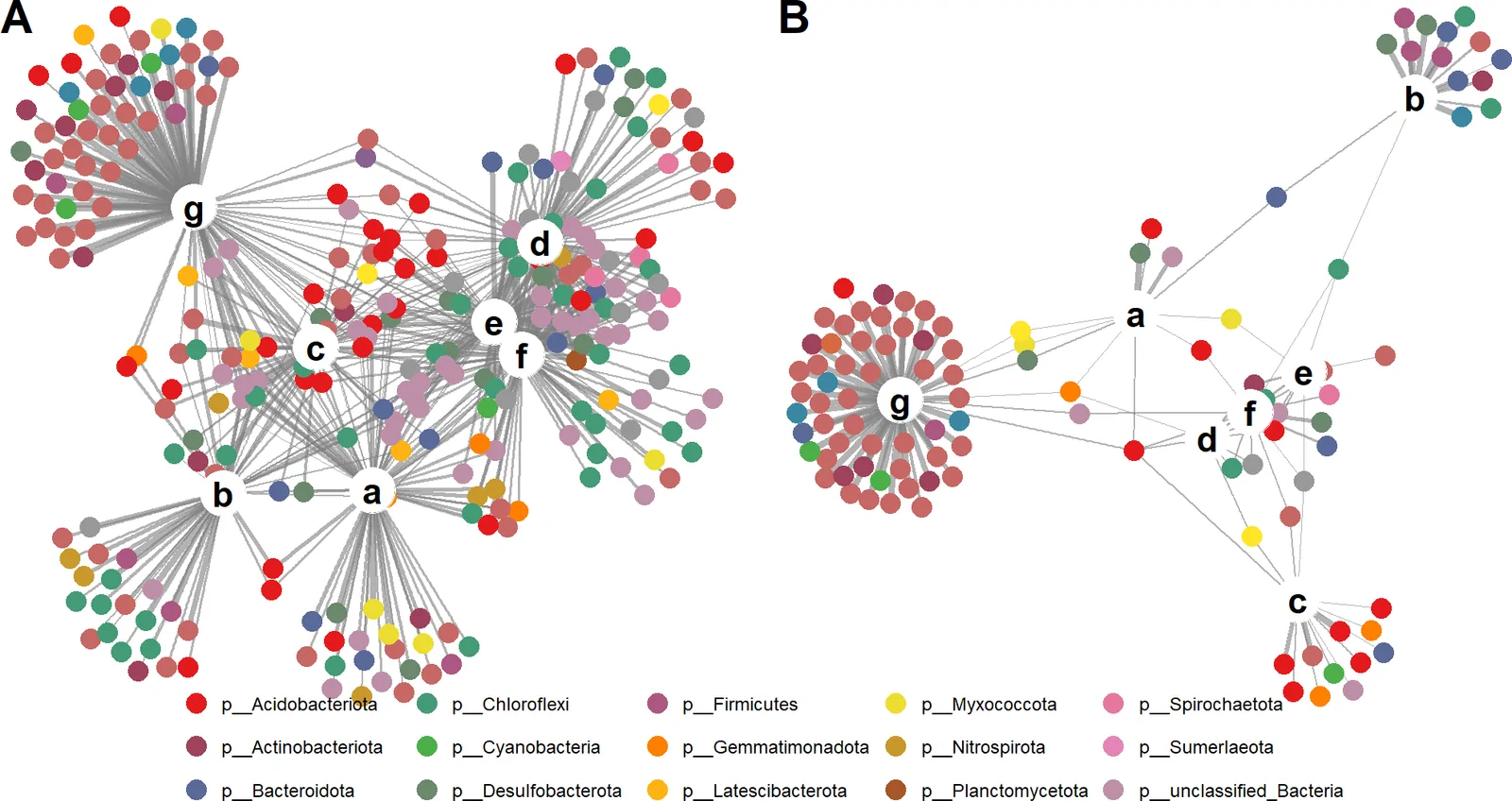
Bipartite networks display sensitive ASVs in RCF and RM as determined using indicator species analysis ([**A**] RCF; [**B**] RM).

**Fig 6:**
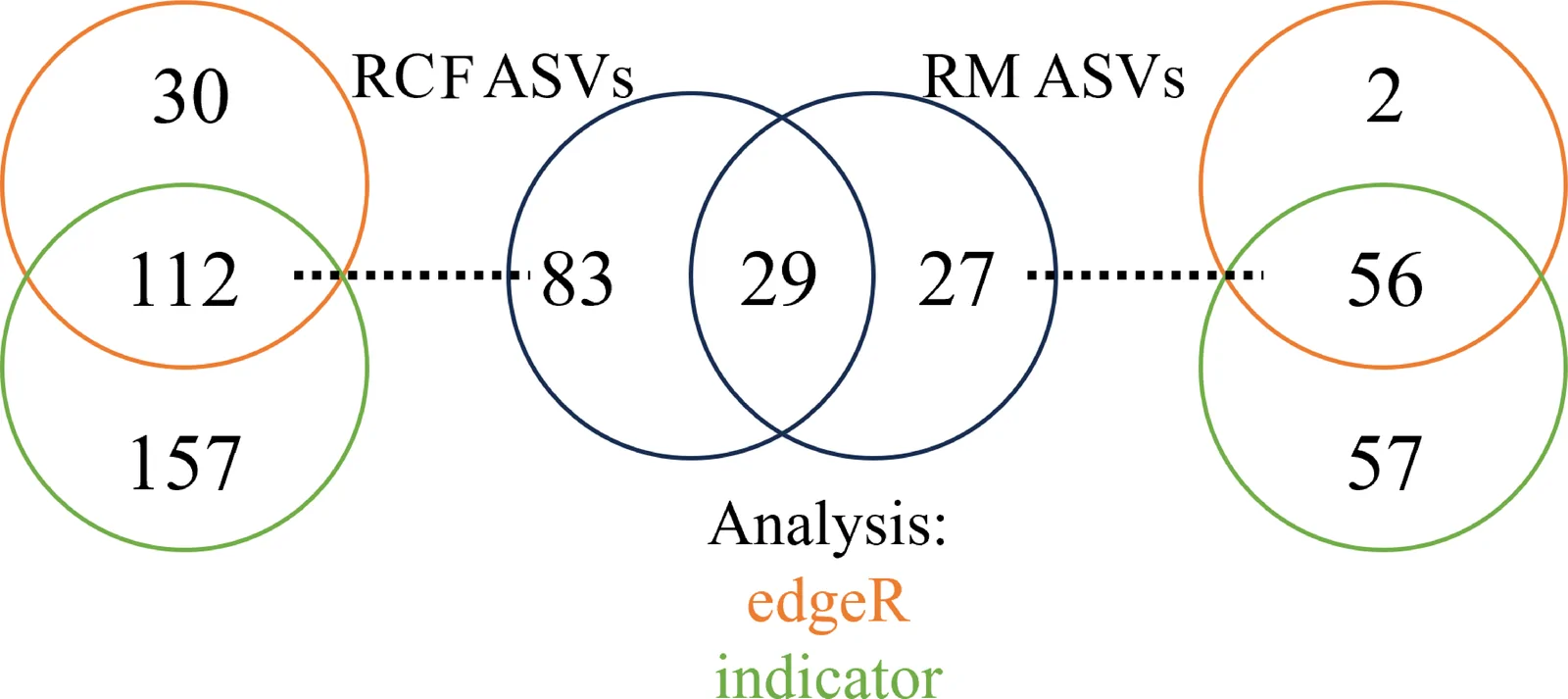
Venn diagrams show the number of ASVs responding to farming stages identified with indicator species analysis (green) and by edgeR (orange). ASVs identified by both methods were defined as sensitive ASVs.

Indicator species analysis was used to identify the individual exhibiting changes in microbial community abundance across different farming stages under two farming modes. The results showed that a large number of ASVs were shared among farming stages in RCF. Moreover, RCF had more stage-specific sensitive ASVs than RM, especially in stage g (Fig. 5). Sensitive ASVs were defined as the intersection of ASVs identified by indicator species analysis and edgeR. Ultimately, 112 and 56 sensitive ASVs were identified in RCF and RM, respectively (Fig. 6). As an approximation of the “effect size” for the farming stage on microbial communities, we found that these RCF- and RM-sensitive ASVs accounted for 1.53% and 0.76% of the soil community sequences, respectively. This is consistent with the previous conclusion that farming stages have different effects on RCF and RM communities.

**Fig 7:**
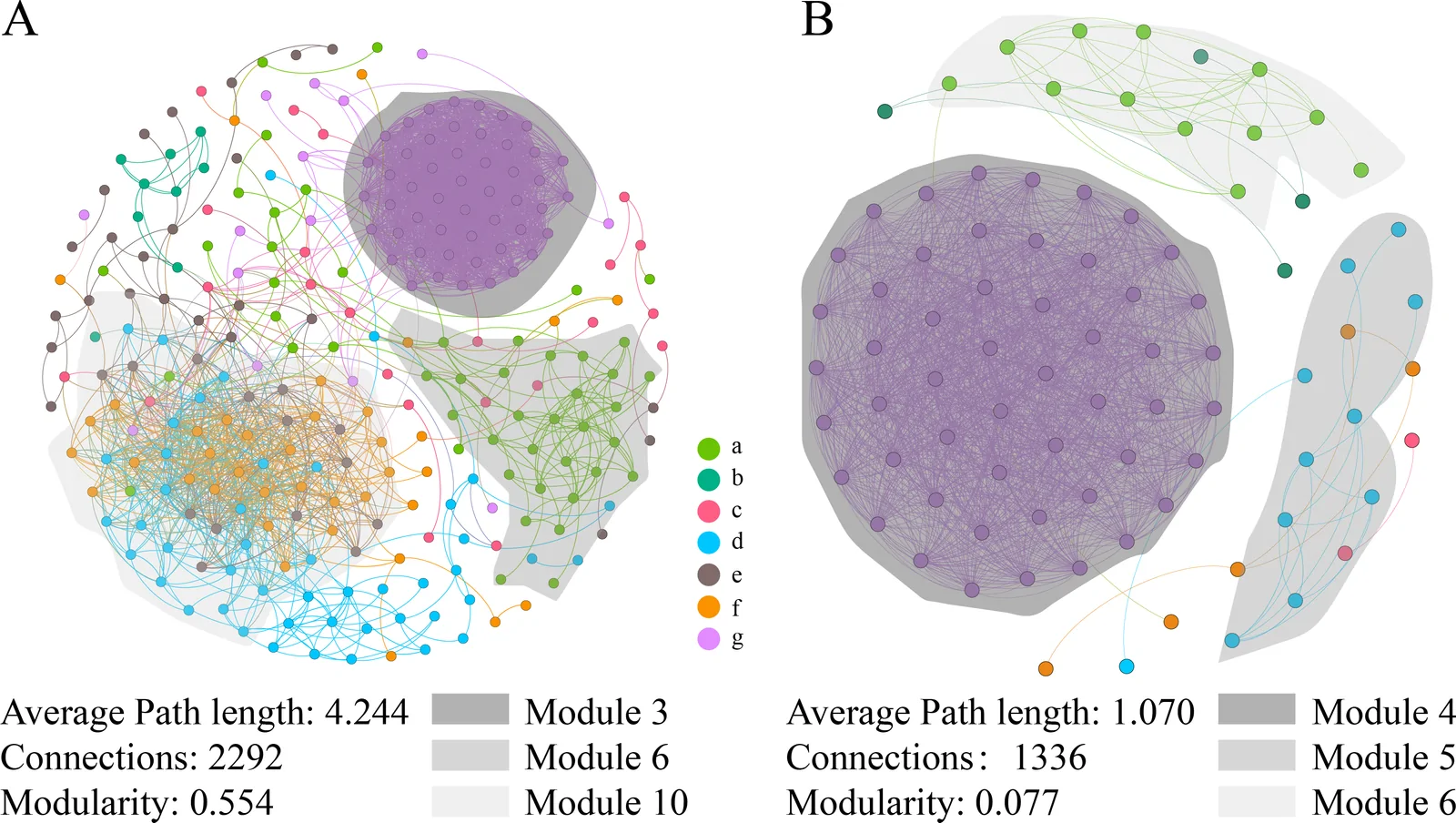
The co-occurrence networks of indicator species ASVs in different farming modes. ([**A**] RCF; [**B**] RM).

The union of the results from the indicator species analysis and edgeR analysis was used to identify indicator species for the co-occurrence network analysis (Fig. 7). The results showed that RCF had a longer average path length (4.244) than RM (1.077), indicating a higher connectivity in RCF. Moreover, the modularity of RCF (0.544) was greater than that of RM (0.077), suggesting the formation of multiple tightly connected modules within RCF.

### Correlations of the sensitive ASVs with soil properties

**Fig 8:**
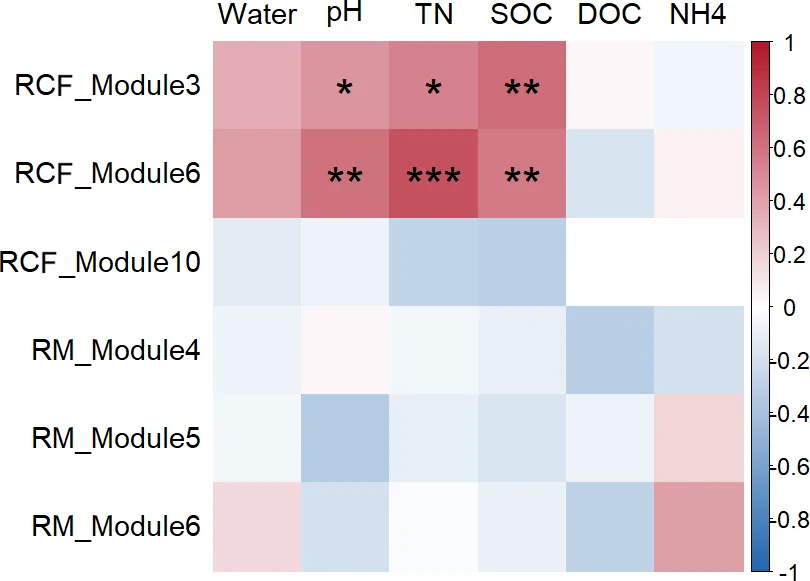
Spearman correlation analysis of the microbial modules and soil properties in two modes. * *P* < 0.05, ** *P* < 0.01 and *** *P* < 0.001.

Spearman correlation analysis revealed relationships between the modules and soil properties (Fig. 8). The results showed that modules 3 and 6 in RCF were significantly positively correlated with pH, TN, and SOC (*P* < 0.05). In contrast, the modules in RM were negatively correlated with most soil properties, although no significant correlations were detected.

## DISCUSSION

### Variations of the soil properties in RCF and RM

Our study demonstrates that RCF significantly increases the content of TOC and TN in topsoil, which is consistent with previous reports (25, 26). In RCF, unconsumed artificial feed and crayfish excretion serve as highly efficient supplementary sources of organic material (27). Upon decomposition by soil microbes, this protein-rich organic carbon directly contributes to the soil pools of SOC and TN (15, 28). The stable pH level observed in RCF is likely attributable to its higher organic matter content, which enhances the buffering capacity of soil and mitigates acidification pressure (29). The NH_4_^+^-N content in RCF was significantly higher than that in RM. This can be explained by exogenous organic carbon inputs and prolonged anaerobic conditions created by flooding (25). These conditions provide increased substrates for ammonification while simultaneously inhibiting nitrification, thereby amplifying the accumulation of NH_4_^+^-N, particularly during the crayfish farming stage (30). Conversely, the simplified ecosystem of the RM relies solely on chemical nutrient inputs, making soil nutrients highly vulnerable to leaching and volatilization (31).

### Different microbiome structure in RCF and RM

Our study demonstrated that microbial richness (Chao1) was influenced by the farming stage. This finding suggests that the farming stage drives the selection of distinct microbial assemblages. In RCF, the farming stage involved alterations of soil moisture, temperature, and other environmental factors through processes such as “flooding,” “seasonal variation,” and “feed inputs” (20). Excessive accumulation of soil nitrogen and moisture stress may lead to a decline in soil microbial community richness and diversity (32). Additionally, our results showed that farming mode significantly influences soil microbial diversity (Shannon index), consistent with previous studies (15, 33, 34).

RCF formed a distinct soil microbial community compared to RM, which is consistent with the results of previous studies (14, 19). Variations in microbial community structure are correlated with changes in microbial functions (23). The RCF exhibited a higher prevalence of sulfur-related processes and a lower prevalence of nitrogen-related processes, which aligns with previous findings (35, 36). Overutilization of the RCF has also been shown to reduce the abundance of functional nitrogen-cycling bacteria in the soil (20, 37). Additionally, the high nutrient content in RCF may lead to the loss of the *nif*H gene, thereby limiting the potential for soil nitrogen fixation (38). In conclusion, the deficiency of nitrogen cycling functions in RCF may be associated with substantial nitrogen input and overutilization during the cultivation process. While FAPROTAX analysis reflects biogeochemical cycling based on taxonomy, direct assessment through functional gene detection is required in future studies.

### Variations in stage-sensitive microorganisms and co-occurrence networks

The distribution of stage-sensitive ASVs, which are key components of the network structure, reveals that microbial community assembly is driven by distinct farming stages. Stage-sensitive ASVs play a pivotal role in shaping microbial networks during the farming process (24). This conclusion was further supported by the PCoA results (Fig. 3), which demonstrate clear temporal shifts.

Compared with RM, RCF exhibited a greater number of nodes and connections. This finding was consistent with the results of the bipartite plot, which indicated that microorganisms in RCF show a stronger response to farming stages. This may be caused by the stable resource supply and clear niche differentiation across stages (20, 21). The higher modularity observed in RCF suggests that its microbial community has greater resistance to disturbance than that of RM, consistent with previous studies (37). Additionally, the co-occurrence network further indicated that the microbial community in the later stage of crayfish farming formed a stable module, suggesting higher stability and a strong buffering capacity (39). This can be explained by long-term flooding, which causes a dramatic shift in the soil redox status and moisture conditions, leading to a severe disruption of the microbial structure (40–42).

Differences in stability mechanisms between the RCF and RM modes were further elucidated by key network modularity indices, including average path length, network diameter, and modularity (43, 44). Our study revealed greater redundancy at various farming stages of RC, thereby enhancing the understanding of redundancy in RCF.

### Relationship of soil properties and soil microbial community

Correlation analysis between sensitive ASV modules and soil properties further revealed differences in microbe-environment interaction mechanisms under two farming modes. Soil pH, TN, and SOC were the key factors influencing microbial structure in the RCF system, indicating that these modules in RCF perform fundamental ecological functions related to nitrogen transformation and organic matter turnover. This may serve as the ecological foundation for material cycling in RCF. In RCF, the accumulation of crayfish feces and the input of artificial feed significantly increased soil organic matter (45), while prolonged flooding led to incomplete decomposition of soil organic matter, disrupting soil nutrient balance (46). In contrast, the modules in RM exhibited a negative correlation trend with most soil properties, none of which reached statistical significance, reflecting that the relationship between the sensitive microbial community and soil nutrients under RM was weakened by environmental fluctuations. This loose association is consistent with the extremely low modularity level of the RM network, making it difficult to form a stable environment-microbe feedback system (47). In summary, RCF enhances the soil environment, forming functional microbial modules closely coupled with nutrient cycling, and promotes modular differentiation within the network. A positive feedback loop of “environmental stability—functional differentiation—modular synergy” is established in RCF, which may be the microbiological basis for maintaining soil quality and microbial community stability in this system.

## Data Availability

The DNA sequences of the 16S rRNA gene were deposited in the National Center for Biotechnology Information (NCBI) under Bio Project number PRJNA1173339. The data that support the findings of this study are available from the corresponding author upon reasonable request.
